# Global Trends in Natural Biopolymers in the 21st Century: A Scientometric Review

**DOI:** 10.3389/fchem.2022.915648

**Published:** 2022-07-07

**Authors:** Yitao Sun, Yinping Bai, Wenlong Yang, Kangmin Bu, Sikander Khan Tanveer, Jiangbo Hai

**Affiliations:** ^1^ College of Agronomy, Northwest A&F University, Xianyan, China; ^2^ College of Life Sciences and Engineering, The Southwest University of Science and Technology, Mianyang, China; ^3^ National Agriculture Research Center, Islamabad, Pakistan

**Keywords:** natural biopolymer, quantitative visualization, bibliometric analysis, VOS viewer, knowledge mapping, Citespace

## Abstract

Since the 21st century, natural biopolymers have played an indispensable role in long-term global development strategies, and their research has shown a positive growth trend. However, these substantive scientific results are not conducive to our quick grasp of hotspots and insight into future directions and to understanding which local changes have occurred and which trend areas deserve more attention. Therefore, this study provides a new data-driven bibliometric analysis strategy and framework for mining the core content of massive bibliographic data, based on mathematical models VOS Viewer and CiteSpace software, aiming to understand the research prospects and opportunities of natural biopolymers. The United States is reported to be the most important contributor to research in this field, with numerous publications and active institutions; polymer science is the most popular subject category, but the further emphasis should be placed on interdisciplinary teamwork; mainstream research in this field is divided into five clusters of knowledge structures; since the explosion in the number of articles in 2018, researchers are mainly engaged in three fields: “medical field,” “biochemistry field,” and “food science fields.” Through an in-depth analysis of natural biopolymer research, this article provides a better understanding of trends emerging in the field over the past 22 years and can also serve as a reference for future research.

## 1 Introduction

The growing global focus on issues such as sustainable development means that these non-biodegradable materials will eventually be phased out. The growing awareness of forecasts for future shortages of fossil resources has led to a growing interest in using environmentally friendly polymers, thereby encouraging the development of biodegradable, non-toxic alternative materials (McDevitt et al., 2017). The rational selection of starting materials is one of the most critical aspects of the materials development process (Kitsara et al., 2015).

Natural biopolymers have attracted much attention among the various polymers currently being produced due to their easy availability, low cost, biocompatibility, unique properties, and wide range of uses (Koh et al., 2015; Huber et al., 2017). Many industries, such as clothing, food, medical, and chemical industries, use materials with specific properties, such as some naturally occurring biopolymer materials: spider silk, silk, etc., as clothing, showing excellent mechanical properties (Leite Lima et al., 2013; Sionkowska and Planecka, 2013; Vishwanath et al., 2016); soy protein, which can make biodiesel with good combustion performance, excellent environmental performance and regeneration performance to replace ordinary fuel (Khansari et al., 2012). Lignin, as the third most abundant natural polymer material, can be used for production as a by-product of the pulp industry. (An et al., 2017).

In the past 10 years, the natural biopolymers that have attracted much attention from all walks of life include nanoparticle polymers (Oliveira et al., 2020). The production of this polymer biocomposite is mainly through electrospinning technology, and the quality of the product depends on the size, content, and structure of the nanoparticles (Vieira et al., 2011). The biodegradable and water-soluble natural polysaccharide (sodium alginate) has been widely used as a building material due to its easy availability and non-toxicity (Jain et al., 2021). The most prominent features are high porosity and stability at different temperatures and pH values (Abriat et al., 2021). Silk fibroin (Shivananda et al., 2020), as a biocompatible natural polymer, has biodegradability, elastic mechanical properties, and processing ability and is suitable for peripheral nerve conduits. In the future, it is expected that nerve regeneration of peripheral nerve conduits can be achieved by the physical guidance of damaged nerves and nerve-guiding conduits (Farokhi et al., 2017). In order to take advantage of the unique properties of these multifunctional natural biopolymers and realize their full potential, scientists continue to study them and try to derivatize them.

Bibliometric analysis is a new data-driven method that applies statistical methods to scientific results (Donthu et al., 2021; Tan et al., 2021) and is widely used in research trend detection, institutional cooperation analysis, national cooperation analysis, and changes in subject areas, with knowledge-oriented quantitative functions (Sun et al., 2019; Kampf et al., 2020). By filtering and processing a large amount of information, the correlation between various data can tap the potential value of knowledge. Since mathematics, statistics, and computer science are the foundation of its disciplines, bibliometrics can provide intuitive data analysis and accurate insights into the progress of scientific research (Durieux and Gevenois, 2010; Bornmann and Mutz, 2015). This study carried out a rigorous quantitative analysis and statistical demonstration based on mathematical models. The purpose of this study was to use the analysis tools that come with Web of Science (WOS), VOS Viewer software (van Eck and Waltman, 2010), and CiteSpace software (Chen, 2006) to carry out the bibliometric analysis. Furthermore, knowledge graphs combine information visualization technology with traditional scientometric citation analysis to visually display the knowledge of a subject or field through data mining, information processing, scientific measurement, and graphic drawing. Using knowledge graphs, one can explore the development and relationships between different pieces of scientific knowledge (Shiffrin and Borner, 2004). Therefore, this study provides a data-driven bibliometric analysis strategy and framework for mining the core content of massive literature data. By using the current mainstream bibliometric software and methods, this study comprehensively reviews the research progress of natural biopolymers from germination to peak from 2000 to 2021, introduces the research background of natural biopolymers, and puts forward original opinions on future opportunities, to help different institutions around the world to cooperate and exchange in the future. First, bibliometric frequency analysis and interaction analysis identified the main subject categories, profiles, cooperation relevance of representative countries and institutions, and mainstream journals. Then, through citation analysis and keyword analysis, we can grasp the trends and laws of dynamic changes in this research field and provide clues for discovering current research hotspots and knowledge gaps. More importantly, knowledge structure analysis and hot front identification provide a holistic view of the field’s evolution and identify more future research opportunities.

## 2 Methodology and Data

### 2.1 Data Source and Retrieval

This article was searched on 17 April 2022 through the Science Citation Index Expanded (SCIE) and Social Sciences Citation Index (SSCI) databases in the Web of Science Core Collection for articles on natural biopolymers from the 21st century. The reason why the WOSCC database was selected for retrieval is that compared with other databases (PubMed and Scopus), it contains more than 10,000 subject areas such as environment, medical care, ecology, and agriculture, with international authority, great influence, high quality, and long history of research data (Harzing and Alakangas, 2016; Mongeon and Paul-Hus, 2016; Zhao, 2017). In order to avoid bias due to the daily update of the WOSCC database, the articles required for the search were carried out within one day on 17 April 2022, and articles published from 1 January 2022, were excluded because, from this period, any collection from that year will include incomplete bibliometric data for that year. In this study, the Web of Science Core Collection (Science Citation Index Expanded, SCIE and Social Sciences Citation Index, SSCI) database is used as the data source, and the retrieval formula is: {TI = [(natural*) and (“biopolymer” or “biopolymers” or “biological polymer” or “biological polymers”)]} OR {AB = [(natural*) and (“biopolymer” or “biopolymers” or “biological polymer” or “biological polymers")]}; search time range: (time = 1 January 2000—31 December 2021). A total of 3,507 articles were retrieved using this search method, and the retrieved literature types included 2,561 articles, 835 reviews, 84 conference proceedings, and 27 other articles. These articles come from 101 countries, 200 institutions, 133 research directions, 200 published journals, and 200 authors. Most of the literature categories belong to polymer science, chemistry multidisciplinary, biochemistry molecular biology, materials science multidisciplinary, and chemistry applied. The retrieved literature records are downloaded and saved in the “full record and cited references” format as the main file and as a sample of the dissertation analysis data.

### 2.2 Activity Index and Attractive Index

Referring to previous studies, we employed two indexes, the activity index (AI) and the attractive index (AAI) to evaluate the relative effort devoted by a country to a research field and the relative impact made by a country in terms of citations of its publications. The AI is an indicator of the relative effort a country puts into a research area, while the AAI shows the relative impact a country has had in attracting citations through its publications (Shen et al., 2018; Huang et al., 2020).

The applications and transformations of AI and AAI are as follows:

Activity index (AI):

$$AI_{i}^{t}=\frac{P_{i}^{t}/\sum P}{TP^{t}/\sum \mathrm{TP}}.$$ (1)

Attractive index (AAI):

$$\mathrm{AA}I_{i}^{t}=\frac{C_{i}^{t}/\sum C}{TC^{t}/\sum \mathrm{TC}}.$$ (2)

In formula (1), $AI_{i}^{t}$ is the activity index of country i in year t, $P_{i}^{t}$ is the number of relevant articles in the field of natural biopolymers published by country i in year t, ∑P is the articles in the field of natural biopolymers published by country i during the publication period , $TP^{t}$ is the total number of publications in the field of natural biopolymers in year t, and ∑TP is the total number of publications in the field of natural biopolymers in a period. Similarly, in formula (2), $\mathrm{AA}I_{i}^{t}$ is the attractiveness index of country i in year t, $C_{i}^{t}$ is the number of citations of publications in the field of natural biopolymers in country i in year t, ∑C is the natural biopolymers in country i in a period sum of citations of field publications, $TC^{t}$ represents the number of citations in the field of natural biopolymers worldwide in year t, and ∑TC represents the total number of citations in the field of natural biopolymers in the same period as ∑C .

AI = 1 and AAI = 1 represent the global average for research work and academic impact in natural biopolymers, respectively. AI > 1 or AI < 1 means a country’s research effort is above or below the global average, and AAI >1 or AAI <1 means a country attracts more or more minor citations than the global average.

## 3 Results and Discussion

### 3.1 Characteristics of Publication Outputs

**FIGURE 1:**
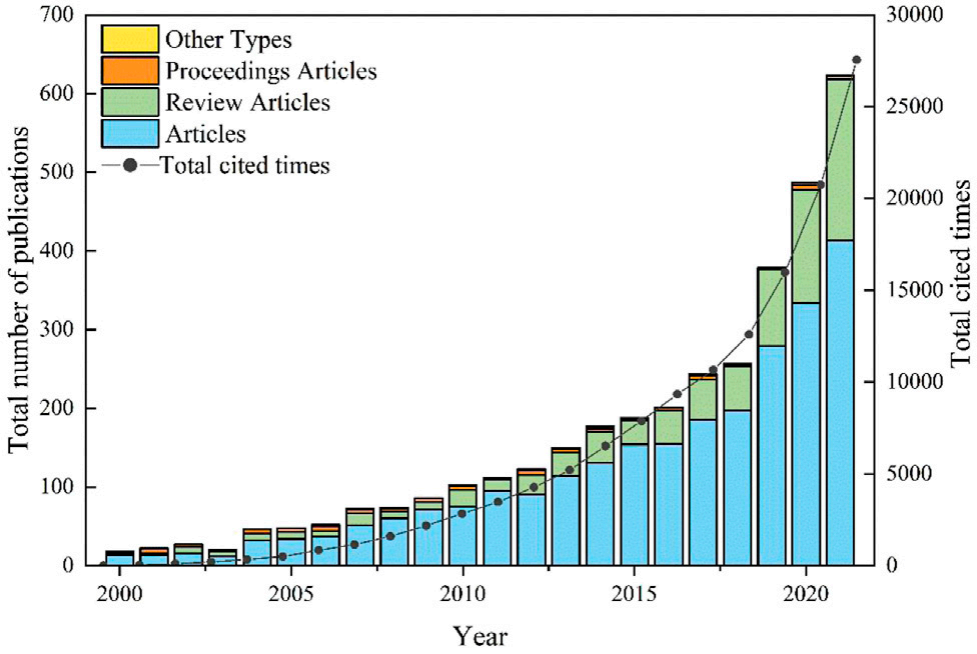
Number of publications and citation frequency from 2000 to 2021.

The annual publication volume of natural biopolymers is divided into three stages (Figure 1). From 2000 to 2002, it was in its infancy. A total of 67 articles were published, with an average annual publication of about 22 articles, 139 citations, and an average annual citation of about 46 times. Although the number of articles at this stage is small, there are still highly cited and high-quality articles. Jeong et al. (2002), according to the law of the transformation of an aqueous polymer solution of a gel to forming *in situ* hydrogels by changing the environmental conditions (temperature and pH), reviewed the introduction of natural or modified natural polymers and N-isopropyl based on acrylamide copolymers and other materials. Mohanty et al. (2002) described that the use of natural fibers and polymers based on renewable resources would solve many environmental problems, adding renewable resource–based biopolymers to fibers, such as green biocomposites, for example, cellulose plastics and polylactic acid will promote the rapid development of green materials in the 21st century.

From 2003 to 2017, it was a period of rapid growth, with a total of 1,694 publications, an average annual publication volume of about 113 articles , 56,948 citations, and an average annual citation of about 3,797 times. In this stage, the research on natural biopolymers is more in-depth, and people are interested in research in this field. The most cited article is Lutolf and Hubbell (2005). The review introduces a new generation of synthetic biomaterials that mimic the regulatory properties of natural extracellular matrix (ECM) and ECM-bound growth factors for therapeutic applications and basic biology research; Nair and Laurencin (2007) gave a review on the synthesis, biodegradation, and biomedical applications of degradable synthetic and natural polymers. By introducing a naturally biocompatible nanomaterial Elo halloysite, Lvov et al. (2016) performed *in vitro* and *in vivo* studies in biological cells and worms to verify the safety of halloysite, as well as the efficient adsorption of mycotoxins in animal stomachs. Lutz et al. (2016)reviewed modern strategies to develop complex synthetic substances, such as controlled radical polymerization, supramolecular polymerization, or stepwise synthesis, to synthesize polymers with precisely controllable molecular structures.

From 2018 to 2021, it is an explosive growth stage, with a total of 1,746 articles published, with an average annual publication volume of about 437 articles , a total of 76,852 citations, and an average annual citation of about 19,213 times. Annually published articles reached a peak of 623 articles, with 27,546 citations. This stage is about 19.9 times the average annual publication volume of the first stage and 3.9 times the average annual publication volume of the second stage. It can be seen that while academic achievements are increasing day by day, researchers’ understanding of natural biopolymers is getting deeper and deeper, and research in this field has become a research hotspot and research trend for scholars and institutions at present and even in the future.

### 3.2 Analysis of Subject Categories, Countries/Regions, Institutions, Highly Cited Journals, and Highly Cited Articles

By analyzing subject categories, countries/regions, institutions, journals, and highly cited articles, this section enables researchers to identify influential and representative subject, countries, institutions, journals, and articles in the field, thereby helping them find research collaborations in this field partner.

#### 3.2.1 Subject Categories

The top 10 discipline categories are shown in Table 1, including polymer science (765 articles, accounting for 21.81% of the total), chemistry multidisciplinary (518 articles, 14.77%), biochemistry molecular biology (437 articles, 12.46%), materials science multidisciplinary (518 articles, 14.77%), 430 articles, 10.27%), chemistry applied (360 articles, 9.12%), chemistry physical (320 articles, 8.30%), food science technology (291 articles, 7.07%), engineering chemical (248 articles, 6.73%), materials science biomaterials (236 articles, 6.53%), and biotechnology applied microbiology (229 articles, 5.86%).

**TABLE 1:** Article output in 10 subject categories of natural biopolymer research.

Subject category	2000–2002	2003–2017	2018–2021
Polymer science	12	325	428
Chemistry multidisciplinary	7	244	267
Biochemistry molecular biology	13	214	210
Materials science multidisciplinary	5	202	223
Chemistry applied	2	148	210
Chemistry physical	5	163	152
Food science technology	2	110	179
Engineering chemical	5	126	117
Materials science biomaterials	1	120	115
Biotechnology applied microbiology	2	133	94

In Table 1, according to the aforementioned article quantity trend analysis, the period is divided into 2000–2002, 2003–2017, and 2018–2021. The number of various publications reflects the development trend of natural biopolymers in different fields. The number of publications in various fields increased significantly between 2000 and 2017, with the most significant increases in chemistry applied, food science technology, materials science biomaterials, and biotechnology applied microbiology. At different times, from issue 1 to issue 2, the number of publications in all categories increased significantly. However, from the second to the third issue, slow growth in the number of publications in each category can be observed.

**FIGURE 2:**
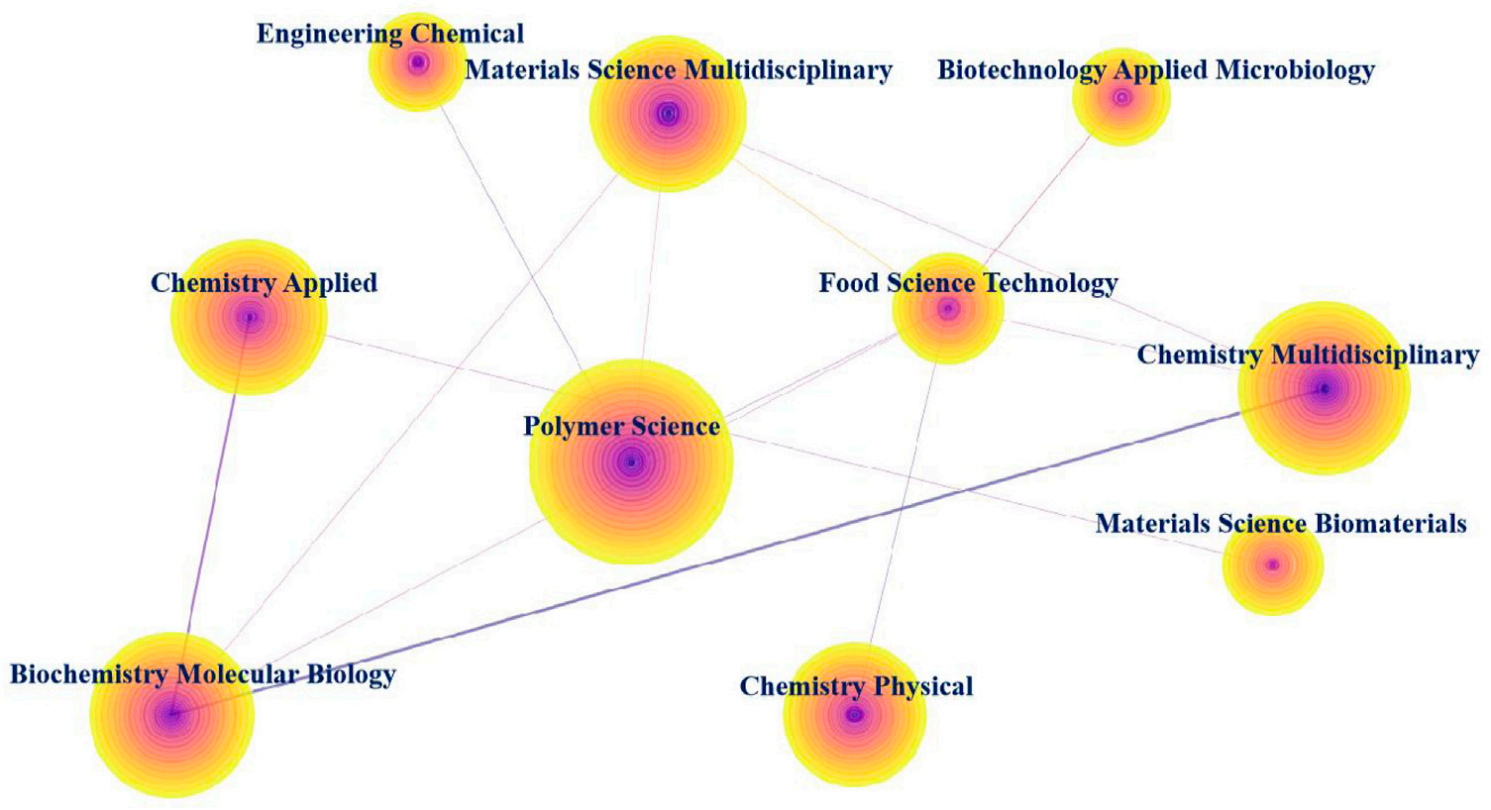
Visualization of the co-occurring subject categories network. (Nodes represent subject categories. The size of a node is proportional to the literature number of the subject category. The links represent the co-occurring relationship between different subject categories. The color of the rings and links corresponds to the year. The purple rings indicate high centrality.)

Co-occurrence category analysis is used to study interdisciplinary linkages, and the construction of subject-related networks can reveal the intrinsic links between different subject categories. The subject area co-occurrence map of natural biopolymers was produced using CiteSpace software (Figure 2). We found that natural biopolymer research is a multidisciplinary and interdisciplinary field, mainly involving polymer science, chemistry, multidisciplinary, biochemistry molecular biology, materials science, multidisciplinary, chemistry applied, chemistry physical, food science technology, engineering chemical, materials science biomaterials, and biotechnology applied microbiology, and many other disciplines. Figure 2 shows that the top three categories of natural biopolymer research are polymer science, chemistry multidisciplinary, biochemistry, and molecular biology. Polymer science emerged as a medium connecting many different disciplines, including chemistry multidisciplinary, and materials science disciplines. The relationships between other disciplines such as chemistry multidisciplinary, engineering chemical, and chemistry applied were mainly established after 2003. Biotechnology applied microbiology has relatively few connections with other disciplines. This result reflects the far-reaching impact of natural biopolymers, as it shows that conducting global research requires concerted efforts from different fields.

#### 3.2.2 Country/Region

**FIGURE 3:**
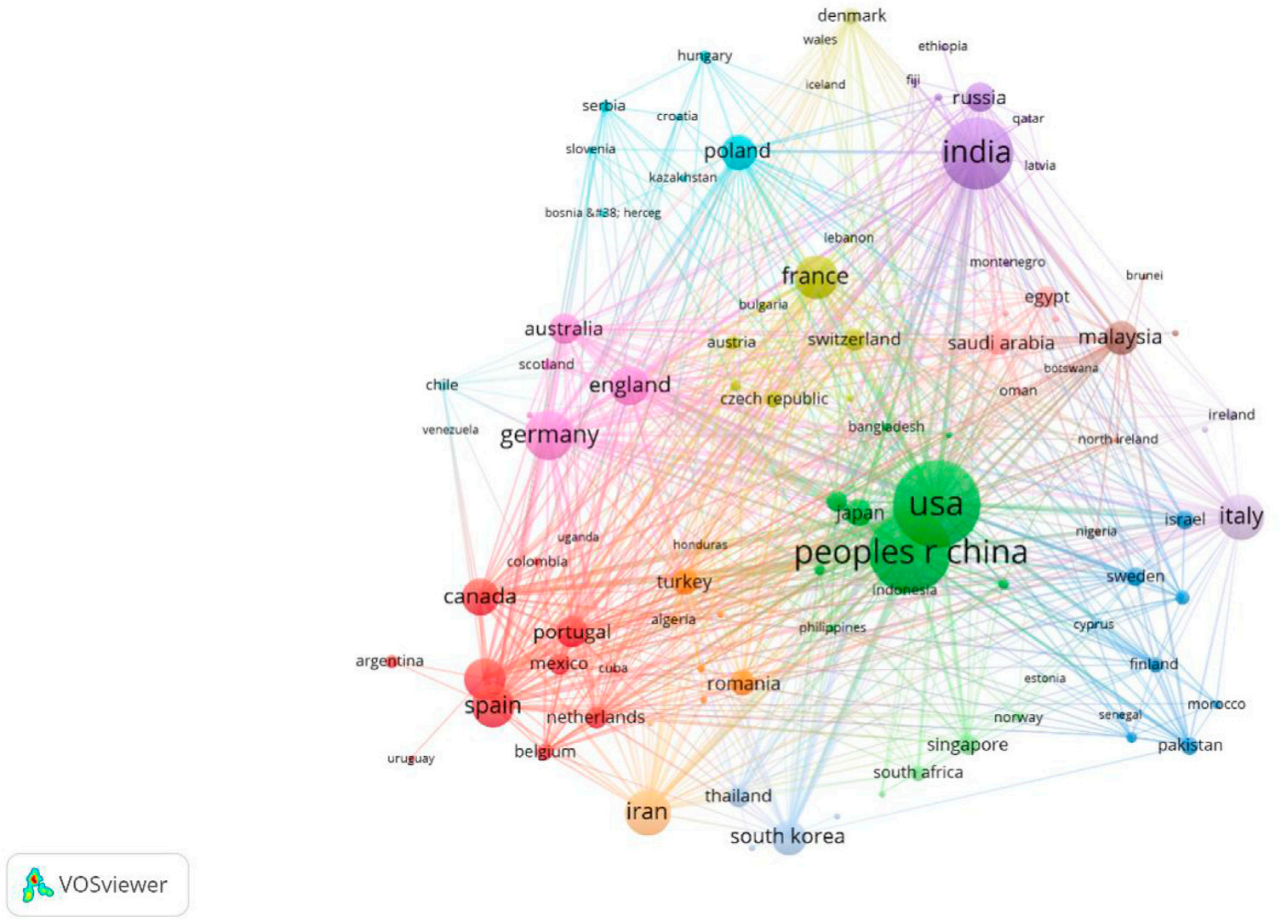
Country/region cooperation knowledge graph (frequency ≥1).

In order to clarify the major countries and regions involved in the field of natural biopolymers and international cooperation, considering the impact of citations on the results, compared with co-occurrence analysis, the literature coupling method (Hassan et al., 2020) can better demonstrate the complex relationship between elements, mining highly consistent unit clusters helps scholars who are engaged in research in the same field to find potential partner countries (Boyack and Klavans, 2010). Therefore, to deepen the analysis of country/region, we adopted a literature coupling method. So far, 101 countries in the world have contributed to the field of natural biopolymers. In order to further explore the cooperation between countries, statistics of the top 10 countries in this field (Table 2), the countries relevant to the research in this field were subjected to literature coupled with visual analysis and mapping using VOS Viewer software (Figure 3). In Figure 3, each node represents a different country, the size of the node represents the country’s scientific output, and the color and connecting line of each node represent a diverse set of co-creation matrices based on the corresponding country. The connections between each country represent the cooperative relationship of each country, and the thickness of the line indicates the degree of cooperation. The stronger the connection between the two nodes, the deeper the connection and cooperation between the two countries.

**TABLE 2:** Top 10 most productive countries in terms of relevant articles.

Country	Ps	Percentage (%)	TC^a^	TC/P^b^	H-index
United States	602	17.17	33,188	55.13	91
China	538	15.34	15,962	29.67	58
India	432	12.32	16,896	39.11	65
Germany	213	6.07	10,749	50.46	49
Iran	188	5.36	6,752	35.91	43
Italy	186	5.30	5,811	31.24	42
France	172	4.90	11,161	64.89	49
Spain	170	4.85	8,100	47.65	43
Brazil	158	4.51	4,496	28.46	30
England	145	4.13	7,500	51.72	42

Note: Ps: the total number of articles published. TC^a^: the total citations for a country. TC/P^b^: average number of citations per paper for a country. h-index: according to Hirsch (2005): A scientist has index H if H of his/her Np articles have at least H citations each, and the other Np-H, articles have no more than H citations each, in which Np is the number of articles published during n years. A higher-index indicates greater academic impact.

According to the statistics of the top 10 countries/regions by the number of published articles (Table 2), the United States has the most significant number of articles published in this field, with 602 articles , accounting for 17.17% of the global total, with a TC^a^ value of 33,188 times and a TC/P^b^ value of 55.13. The h-index value is the largest at 91, indicating that it has many high-quality articles; followed by China with 538 articles, accounting for 15.34% of the global total, with a TC^a^ value of 15,962 times, a TC/P^b^ value of 29.67, and the h-index value is 58; India publishes 432 articles, accounting for 12.32% of the global total, the TC^a^ value is 16,896 times, the TC/P^b^ value is 39.11, and the h-index value is 65. These three countries have representative and authoritative research in this field, with 1,183 articles published, accounting for 1/3 of the world’s articles. France has the highest TC/P^b^ value at 64.89, indicating that the country has many high-quality articles. As shown in Figure 3, Sino-US cooperation is the most frequent, followed by India and South Korea, Germany and Singapore.

**FIGURE 4:**
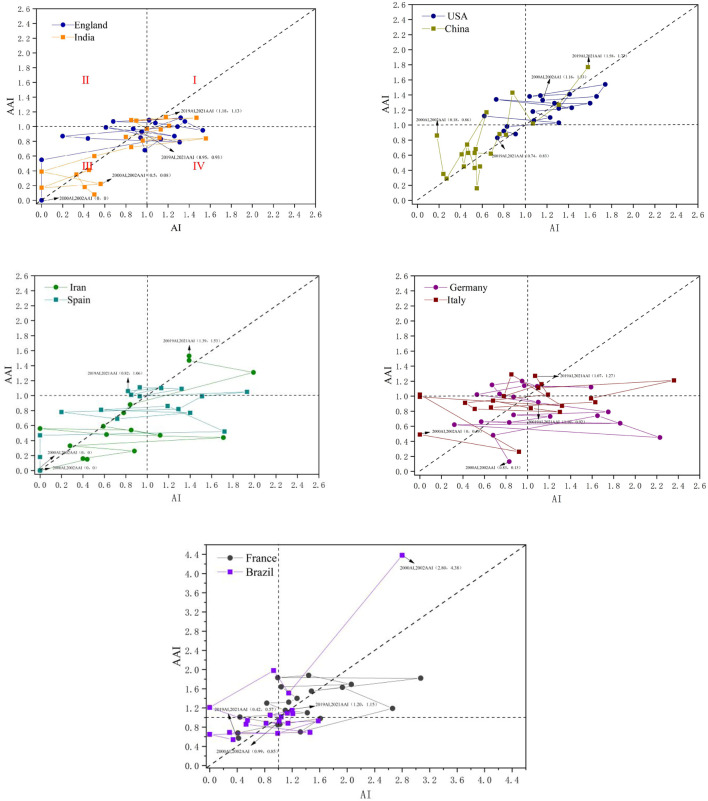
Relational chart of AI and AAI from 2000 to 2021 for 10 countries/regions.

With the development of the times, there are more and more cooperation links between countries; the future research trend in biopolymers will be multinational and diversified. In this part, we use the activity index (AI) and the attractive index (AAI) to evaluate the temporal changes in research and academic impact for selected countries. Considering that there is usually a lag between when an article is published and cited, we set the time horizon for the attraction index to be two years later than the activity index. The starting year of the activity index for a given country is when the country first published an article in the field (Shen et al., 2018). Therefore, the activity and attractive index’s time horizons are 2000–2019 and 2002–2021, respectively. The activity index (AI) and attractive index (AAI) of the top 10 countries with the most publications were calculated (Supplementary Tables S1, S2) and presented in a quadrant diagram (Figure 4). The reference line (y = x) reflects how a country’s research efforts are balanced against the impact it cites in natural biopolymer research. These selected countries have undergone four types of evolution. Quadrants 1–4 in Figure 4 represent four different situations, respectively. The points in the first quadrant represent the years in which the country’s activity index (AI) and attractive index (AAI) are both higher than the global average. Dots represent years in which the country’s attractive index (AAI) is higher than the global average, and the activity index (AI) is lower than the global average; the dots in the third quadrant represent the country’s activity index (AI) and attractive index (AAI) are both low years in which the country’s activity index (AI) is above the global average. The attractive index (AAI) is below the global average.

Comparing the performance of countries using the activity index (AI) and attractive index (AAI) shows that, in most years, the United States and France have higher research efforts and academic influence than the global average; research efforts and academic impact in China, Iran, and India are below the global average in most years but have exceeded the global average since 2019, implying a more critical role for developing countries than in the past, along with their research, technology, and economic areas; wait until their ability begins to improve. Research effort and impact in developed countries decreased relatively (United States, Germany, and France) or fluctuated (Italy, England, Spain, and Brazil) over the same period. However, the research on natural biopolymers in developed countries still has significant influence and status. In addition, the United States and China are relatively close to the reference line in terms of the distance between the points representing a particular country and the reference line, which means that their research efforts are balanced against the impact of citations.

#### 3.2.3 Institution

In the same research field, cooperation between institutions can reflect the most productive organizational, professional information, and inter-institutional relationships, and the frequency analysis and bibliographic coupling analysis of institutions are performed through bibliometrics. A total of 200 institutions globally have contributed to the field of natural biopolymers, and the top 10 institutions with the number of publications are counted (Table 3).

**TABLE 3:** Top 10 most productive institutions in terms of relevant articles.

Institution	Ps	Percentage (%)	Country	TCa	TC/Pb	H-index
League of European Research Universities LERU	119	3.39	United Kingdom etc.	5,214	43.82	39
Centre National De La Recherche Scientifique CNRS	101	2.88	France	5,706	56.5	32
Chinese Academy of Sciences	81	2.31	China	3,043	37.57	33
Udice French Research Universities	73	2.08	France	4,776	65.42	31
Indian Institute of Technology System Iit System	66	1.88	India	2,579	39.08	27
Consejo Superior de Investigaciones Cientificas CSIC	58	1.65	China	3,912	67.45	26
Russian Academy of Sciences	54	1.54	Russia	488	9.04	13
Council of Scientific Industrial Research CSIR India	50	1.43	India	2,491	49.82	20
Consiglio Nazionale Delle Ricerche CNR	46	1.31	Italy	1,354	29.43	17
Egyptian Knowledge Bank EKB	46	1.31	Egypt	1,389	30.2	16

**FIGURE 5:**
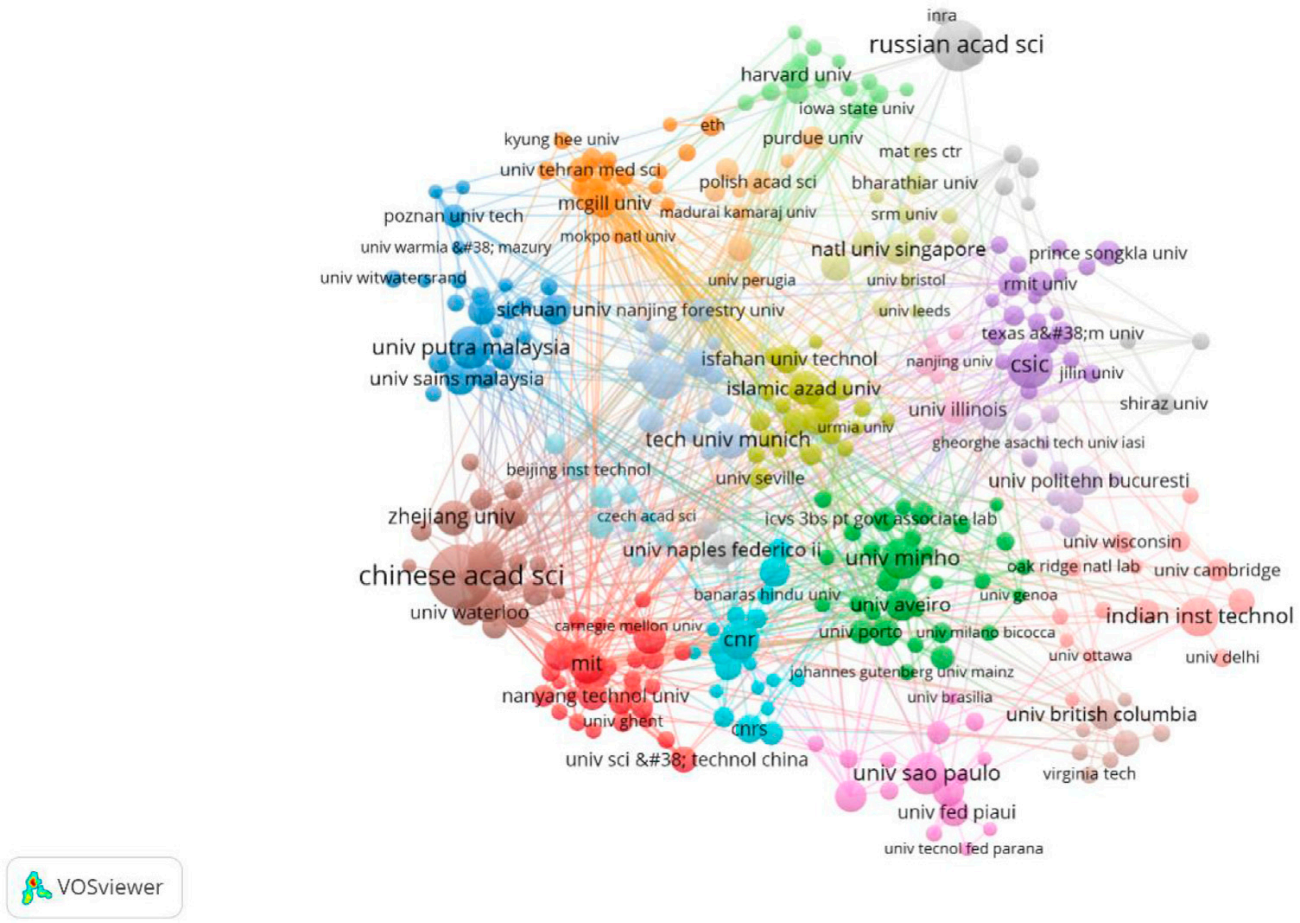
Knowledge map of cooperation between institutions (frequency ≥5).

As demonstrated in Table 3, among the 200 institutions, the League of European Research Universities LERU published the most articles, with a total of 119 articles, accounting for 3.39% of the global total, with a TC^a^ value of 5,214 times and a TC/P^b^ value of 43.82. The h-index value is the largest at 39, indicating that the institution has published many high-quality articles; followed by the Centre National De La Recherche Scientifique CNRS in France (101, 2.88%, 5,706, 56.5, and 32) and the Chinese Academy of Sciences in China (81, 2.31%, and 3,043, 37.57, 33). China’s Consejo Superior de Investigaciones Cientificas CSIC had the highest TC/P^b^ value of 67.45, indicating that the institution has much high quality. Notably, while the United States ranks first for research publications in this field, it does not have a single institution in the top 10 publications. As shown in Figure 5, the Chinese Academy of Sciences is the most productive institution in this field, and it is also the most core and cooperative institution in this field. On the other hand, it can be seen that the various institutions are cooperating with institutions in the same country, and the cooperation of multinational institutions is less. Observing cooperation between institutions will facilitate researchers to find potential partner institutions faster and more accurately and help more institutions carry out cross-border cooperation.

#### 3.2.4 Highly Cited Journals

The top 10 journals are ranked by the number of publications (Table 4). It can be seen that among the top 10 journals with more than 100 articles, there are two journals with more than 100 articles, namely 128 articles from Elsevier’s “International Journal of Biological Macromolecules” and 104 articles from Mdpi’s “Polymers.” In addition, the impact factor (IF) and JCI index of these journals can also measure their value according to their role and status in science communication. The Journal Citation Indicator (JCI) is the average Category Normalized Citation Impact (CNCI) of citable items (articles and reviews) published by a journal over a recent three-year period. Citation impact (JCI > 1) and impact factor (IF > 5) are higher than the average citation impact of similar journals. Among them, “Food Hydrocolloids” has the highest JCI of 2.15, which is 115% higher than the average citation impact of similar journals, and the IF value is 9.169, which indicates that the journal has significant international influence; “International” Journal of Biological Macromolecules, JCI value is 1.38, 38% higher than the average citation impact of similar journals, IF value is 6.737; “Carbohydrate Polymers” JCI value is 1.99, 99% higher than the average citation impact of similar journals, IF value is8.678. The JCI value of “ACS Sustainable Chemistry Engineering” is 1.5, which is 50% higher than the average citation impact of similar journals, and the IF value is 8.471; the JCI value of “Materials Science Engineering C Materials for Biological Applications” is 1.27, which is averagely cited than similar journals. The impact is 27% higher, and the IF value is 6.654; the JCI value of “Biomacromolecules” is 1.60, which is 60% higher than the average cited the impact of similar journals, and the IF value is 6.813; the United States ranks only 4th among the top 10 journals in terms of the number of published articles. Nevertheless, 5 of the top 10 journals (50%) are in the United States. It can be seen that it still has a high research status in this field. It is worth noting that the top 10 journals with published articles are all developed country journals. It can be seen that developed countries have made a lot of research and contributions to natural biopolymers.

**TABLE 4:** Top 10 journals with publication volume.

Journal	Ps	Percentage (%)	Publisher	Region	Total citation	JCI	If
International Journal of Biological Macromolecules	128	3.65	Elsevier	Netherlands	79,247	1.38	6.737
Polymers	104	2.97	Mdpi	Switzerland	27,637	0.83	4.493
Carbohydrate polymers	99	2.82	Isevier sci itd	England	104,570	1.99	8.678
Biopolymers	69	1.97	Wiley	United States	9,962	0.47	2.235
Food hydrocolloids	55	1.57	Isevier sci itd	United States	42,938	2.15	9.169
Journal of Applied Polymer Science	44	1.25	Wiley	United States	68,877	0.7	2.754
Materials	38	1.08	Mdpi	Switzerland	52,459	0.63	3.92
ACS Sustainable Chemistry Engineering	34	0.97	Amer chemical soc	United States	62,273	1.5	8.471
Materials Science Engineering C Materials for Biological Applications	32	0.91	Elsevier	Netherlands	50,537	1.27	6.654
Biomacromolecules	31	0.88	Amer chemical soc	United States	45,724	1.6	6.813

Note: JCI: Journal Citation Indicator (The average JCI, in a category is 1. Journals with a JCI, of 1.5 have 50% more citation impact than the average in that category. It may be used alongside other metrics to help you evaluate journals.); IF: 5-years impact factor, impact factor data from the 2020 edition of Journal Citation Reports^®^ in Web of Science.

#### 3.2.5 Highly Cited Articles

The top 10 most cited articles in natural biopolymers were counted (Table 5) Faruk et al. (2012). “Biocomposites reinforced with natural fibers: 2000–2010,” published in 2012, was cited the most with 2,087 times; followed by Samir et al. (2005). “Review of recent research into cellulosic whiskers, their properties and their application in nanocomposite field” was cited 1,742 times in 2005; Crini (2005) published “Recent developments in polysaccharide-based materials used as adsorbents in wastewater treatment” in 2005, cited 1,510 times. Two of the top 10 cited articles were published in “Progress in Polymer Science” and “Biomacromolecules,” which shows that the journal’s research in this field is representative and authoritative.

**TABLE 5:** Top 10 cited articles and journals.

Title	Journal	Citations	Year	First author
Biocomposites reinforced with natural fibers: 2000–2010	Progress in polymer science	2,087	2012	Faruk, O, Faruk et al. (2012)
Review of recent research into cellulosic whiskers, their properties and their application in the nanocomposite field	Biomacromolecules	1,742	2005	Samir, MASA, Samir et al. (2005)
Recent developments in polysaccharide-based materials used as adsorbents in wastewater treatment	Progress in polymer science	1,510	2005	Crini, G, Crini, (2005)
Sustainable bio-composites from renewable resources: Opportunities and challenges in the green material world	Journal of Polymers and the Environment	1,413	2002	Mohanty, AK, Mohanty et al. (2002)
Biopolymer-based hydrogels as acaffolds for tissue engineering applications: a (Review)	Biomacromolecules	1,165	2011	Van Vlierberghe, S, Van Vlierberghe et al. (2011)
Biomass burning—a review of organic tracers for smoke from incomplete combustion	Applied geochemistry	1,013	2002	Simoneit, BRT, Simoneit, (2002)
Hierarchically porous carbon derived from polymers and biomass: effect of interconnected pores on energy applications	Energy and environmental science	984	2014	Dutta, S, Dutta et al. (2014)
Functional and bioactive properties of collagen and gelatin from alternative sources: a (review)	Food hydrocolloids	973	2011	Gomez-Guillen, MC, Gómez-Guillén et al. (2011)
Natural-based plasticizers and biopolymer films: a (review)	European polymer journal	956	2011	Vieira, MGA, Vieira et al. (2011)
Ionic liquid processing of cellulose	Chemical Society Reviews	927	2012	Wang, H, Wang et al. (2012)

### 3.3 Knowledge Structure and Sub-Fields

**FIGURE 6:**
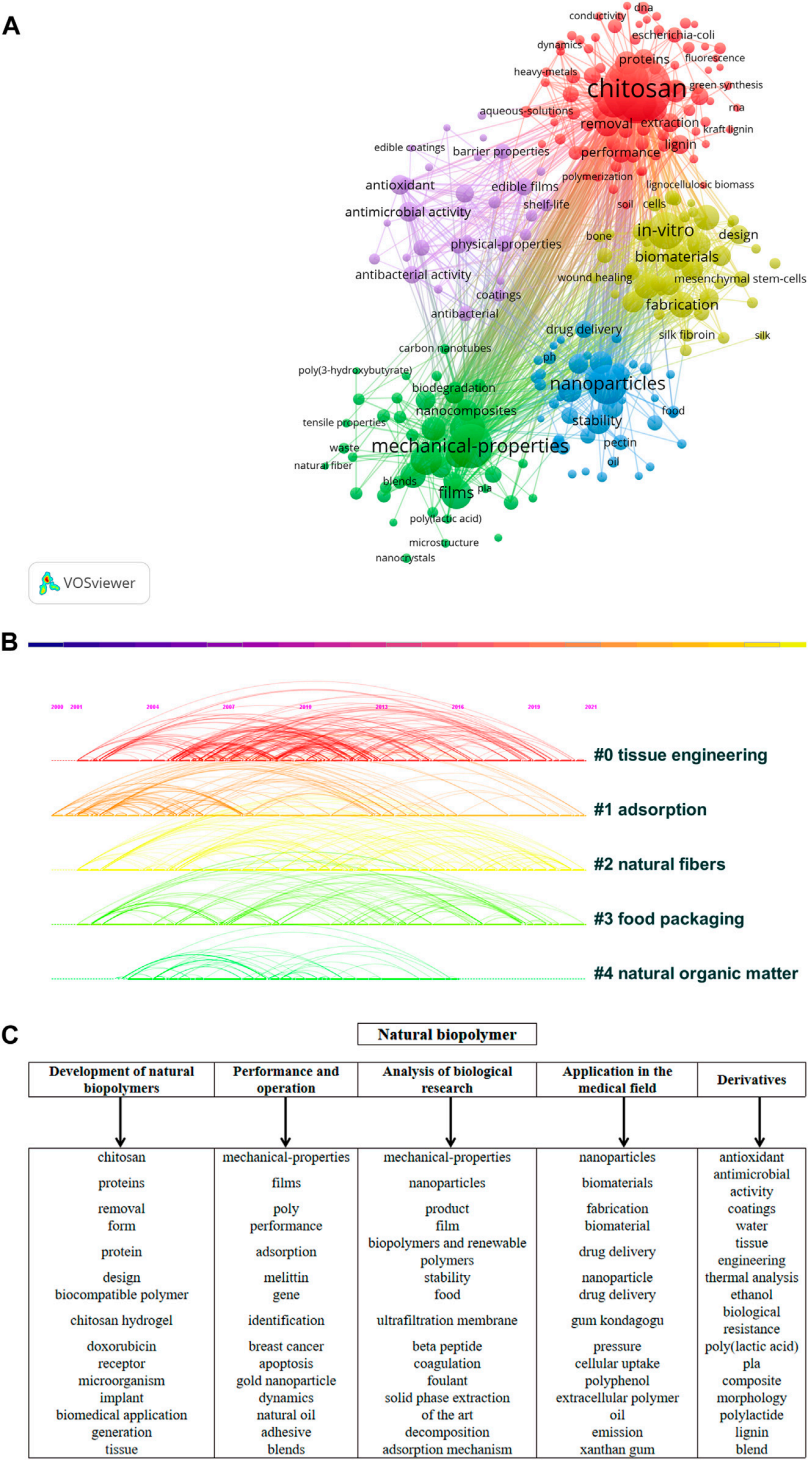
Keyword co-occurrence and clustering knowledge graph. **(A)** Based on VOS viewer software (frequency≥ 20). **(B)** Based on CiteSpace software (top 5 categories). **(C)** Knowledge structure graph.

As the core of a document, keywords are words with substantive meaning condensed from the document’s leading content and have a high guiding role for academic research in a field. Keyword co-occurrence is to mine the relationship between high-frequency keywords. If a particular keyword appears in different documents with high frequency simultaneously, their correlation is very close, representing the hot research in this field (Chen et al., 2015). However, only considering the relationship of the keyword dimension for clustering has certain limitations, ignoring the cooperative relationship between keywords, and it is not easy to interpret the Frontier fields in-depth in terms of classification. Therefore, a bimodal matrix was constructed in this study, and a systematic clustering analysis was performed on high-frequency keywords. In addition to analyzing keywords by VOS Viewer software, this study also introduces Citespace software. Citespace software has diversified, time-based, and dynamic citation visualization analysis, allowing readers to understand the knowledge area of a specific topic through visualization (Chen et al., 2010; Xie, 2015). The keywords in the field of natural biopolymers are visualized and analyzed by VOS Viewer software (Figure 6A), and the CiteSpace software is used to visualize the data related to natural biopolymers from 2000 to 2021. The top five keywords are plotted on the cluster map (Figure 6B).

There are five clusters in the figure, as shown in Figure 6A, indicating that natural biopolymers are mainly divided into five main directions in the current study. The blue clusters represented by the keywords “nanoparticles,” “stability,” and “food,” the green clusters of “mechanical-properties,” “films,” and “blends,” the “*in vitro*,” “yellow clusters for “biomaterials” and “fabrication,” purple clusters for “antioxidant,” “antimicrobial activity,” and “coatings,” and red clusters for “chitosan,” “proteins,” and “removal.”

In summary, the research on natural biopolymers can be divided into five main categories of knowledge structures (Figure 6C), namely, “development of natural biopolymers,” “performance and operation,” “analysis of biological research,” “application in the medical field” and “derivatives.”

#### 3.3.1 Development of Natural Biopolymers

In the past few decades, to reduce the impact of traditional polymers (plastics) on the ecological environment, reduce the consumption of non-renewable resources in the production process, and promote global sustainable development. As a result, the production and application of natural biopolymers have increased substantially over the past few years, making natural biopolymers one of the most promising avenues to achieve sustainable development goals. Natural biopolymers are naturally derived materials, including polynucleotides, polypeptides, and polysaccharides (Wang et al., 2021). The application of natural polymers in biomedicine dates back thousands of years, whereas synthetic degradable polymers only started in the late 1960s (Nair and Laurencin, 2007). Since the 21st century, a series of natural biodegradable polymers have been developed, which are mainly used in polymer science (biofiber-reinforced composites, nanomaterials) (Faruk et al., 2012), biomedicine (tissue engineering, regenerative medicine, gene therapy, and controlled drug release) (Nair and Laurencin, 2007), and biochemistry (hydrogels to enhance bioactivation, cell survival, and tissue formation) (Malda et al., 2013). Notably, new fibers from emerging biopolymers have emerged in recent years, including sulfated polysaccharides (carrageenans and glycosaminoglycans) and tannins and their derivatives (condensed and hydrolyzed tannins, tannins acid) (Reddy et al., 2021). As a water-soluble and complex anionic sulfated polysaccharide, green algae mud is green seaweed cell walls (Lahaye and Robic, 2007). Its carbohydrate composition is complex and changeable, mainly composed of rhamnose, glucuronic acid, iduronic acid, and xylose. The physical properties and pharmacological activities of green algal sulfurized polysaccharides have been systematically studied (Robic et al., 2008; Alves et al., 2010), and a wide range of biological activities, including antibacterial (Gadenne et al., 2015; Berri et al., 2016), antiviral (Shi et al., 2017), and antibacterial coagulation (Zhang et al., 2008). Tannins are low-cost and ubiquitous natural biopolymers (Morisada et al., 2011). They are secondary metabolites of polyphenols of higher plants, mainly found in soft tissues (flakes, needles, or bark) (Hernes and Hedges, 2004; Arbenz and Averous, 2015). After cellulose, hemicellulose, and lignin, tannins are the most abundant compounds extracted from bark, leaves, seeds, and fruits (Khanbabaee and van Ree, 2001). In this field of research, scientists continue to innovate. From the earliest single natural biopolymers (spider silk, insect shells) to today’s various emerging natural biopolymers, it can be seen that the field of natural biopolymers is constantly developing, and scientists are more and more interested in this field, and they have invested much time in research and development. In the future, cross-country and institutional research will become a trend.

#### 3.3.2 Performance and Operation

Natural biopolymers have attracted the attention of researchers because of their unique properties, attractive functions, and abundant resources. The most prominent properties of natural biopolymers include biodegradability, bioactivity, biocompatibility, film-coating ability, high miscibility, environmentally friendly non-toxicity, and are non-allergic (Khan et al., 2017; Verlee et al., 2017; Volova et al., 2018). Biocompatibility is the most important, referring to the interaction between living systems and materials without side effects (Ghasemi-Mobarakeh et al., 2019). For example, advanced functional materials can be made of chitosan, which has antibacterial and antifungal properties (Zhang et al., 2013), electrical conductivity, and electrical activity (You et al., 2017); silk fibroin has superior mechanical properties, good biocompatibility, and biodegradability (Zhou et al., 2017; Ling et al., 2018). Also, scientists (Sell et al., 2009) have exploited the solubility of natural biopolymers in different solvents and different structural behaviors in solutions to fabricate scaffolds and fibers with favorable biological properties. Most of the articles in this field focus on the favorable properties of natural biopolymers and toxicological aspects, unfavorable factors, and how performance indicators change over time. There are fewer reports present that compare various performance parameters of natural biopolymers with other polymers. Still less, many unknown factors still need further systematic study by researchers.

#### 3.3.3 Analysis of Biological Research

Natural biopolymers have been extensively studied in the biological field. For example, natural gums are used as thickeners and suspending agents in various dosage forms and show excellent controlled drug release profiles due to their excellent drug retention (Koyyada and Orsu, 2021); in artificial structural coloring materials, including their applications in biomimetic design and fabrication (Zhang Z. et al., 2021); as proton conductors in bioelectronic devices, such as polysaccharides (chitosan, GAG), proteins and peptides. (Esmaeilzadeh et al., 2018; Jia M. et al., 2021). Agarose, a high-molecular polymer extracted from natural plant gelatin, is a flexible ion-conducting polymer widely used to separate biomolecules (Wang et al., 2011) and solid electrolytes (Finkenstadt, 2005). Although natural biopolymers have many benefits, there are still some deficiencies in integrating biopolymers with bioelectronic devices. For example, the importance of desired delicate patterns is expected to increase as the size of bioelectronic devices becomes smaller, natural biopolymers are heterogeneous when they come from different species or even from the same organism, natural biopolymers with hydration properties in aqueous environments can lead to swelling and potential failure at the interface with other functional electronic components (Jia M. et al., 2021). In this field, the ever-increasing demand will eventually strain the resources of biological raw materials. As a sustainable, green, and efficient biological material, natural biopolymers can reasonably cope with the shortage of some biological resources. But the modification of natural biopolymers is either toxic or does not yield the final desired properties, and there are currently few methods to deal with them, which will require further research by researchers in the future.

#### 3.3.4 Application in the Medical Field

Naturally derived biopolymers are suitable for medical applications due to their biocompatibility, biodegradability, non-toxicity, and ability to adsorb bioactive molecules. In medical applications, natural biopolymer materials can be defined as materials that interface with biological systems for evaluation, treatment, and augmentation of the human body (Nair and Laurencin, 2007), such as bone screws, scaffolds, plates, and multifilament meshes (Vert, 2005). For example, scaffolds are used as a cell support system for seeding cells *in vitro* and stimulate more cells to build up a matrix to build a tissue foundation for transplantation, requiring the use of scaffolds as drug delivery devices or growth factors. This approach combines the scaffold with growth factors, and the body’s implanted cells are recruited around the matrix at the scaffold site to form new tissue. The two approaches are not mutually exclusive and can easily fuse. Naturally derived biopolymers are suitable for medical applications due to their biocompatibility, biodegradability, non-toxicity, and ability to adsorb bioactive molecules (Ripamonti, 2004; Howard et al., 2008). Polyethylene glycol (PEG) is a natural biopolymer (biocompatible hydrophilic polyether) commonly used in medicine to enhance the action of bioactive compounds (Winnacker and Rieger, 2017). The manufacture of synthetic polymers is a controlled procedure involving fixed quantities of ingredients; however, the productivity of natural biopolymers depends on the environment and many physical and other factors. In recent years, natural biopolymers have also received extensive attention in the field of drug delivery (Maghsoudi et al., 2020). Chitosan is a polysaccharide present in shellfish, fungi, annelids, mollusks, and insects, and it is the second most widely distributed natural polysaccharide on earth (Gheorghita et al., 2021). The use of chitosan for drug delivery in the pharmaceutical industry has many advantages, such as the controlled release of encapsulated substances, elimination of toxic substances during development, and improved membrane absorption by mixing cationic chitosan with anionic materials (Prabaharan, 2008). Roy et al. (2020) using natural biopolymers with computer-aided pharmacokinetic modeling conjugates for cancer cell–targeted specific drug delivery will be a focus area for future research. In this field of research, natural biopolymers are mainly used in drugs and medical devices, which can be a good substitute for many refractory and expensive materials. There are few studies on agriculture and other fields, and researchers still need systematic research and interdisciplinary and interdisciplinary collaborative research.

#### 3.3.5 Derivatives

Due to their rich functions, natural biopolymers have derived many valuable products by studying their functions. Electrospinning, resulting from the processing of natural biopolymer materials, can be used as porous fibrous materials for tissue engineering and other biomedical applications (Madruga and Kipper, 2022); glycosaminoglycans, the main components of extracellular matrix in animal tissues, are formed by gel-like matrix, widely used in biomedicine as an anticoagulant, anti-tumor, and anti-inflammatory, and wound healing (Liu et al., 2002; Mizumoto and Sugahara, 2013; Zhou et al., 2016); in electrical stimulation–based bioelectronic devices, natural biopolymer chitosan and carbon nanomaterials materials or conductive polymers can be mixed to form highly conductive composites (Sadeghi et al., 2019); the polyglycolide non-woven fabric-fibrin glue composite matrix formed based on polyethylene glycol has excellent skin closure ability, and it is helpful for biological regeneration of tissues and has been applied in the medical field (Terasaka et al., 2006). In this field, some derivatives are limited by problems such as insolubility in water, fast depolymerization in the human body, and weak antibacterial properties. There are still few studies on functionalizing the structure of natural biopolymers through various chemical modifications. Its derivatives are also mainly concentrated in the medical field, and future research in other fields needs to be further strengthened by researchers.

### 3.4 Research Frontier Identification

Outbreak detection can be used to find keywords of particular interest to the scientific community at a certain time. We can determine the development of keywords and potential research topics (Li et al., 2021), which can be viewed as the appearance time, end time, and keyword strength of keywords, and predict the research trend of future hot areas. Therefore, outbreak keywords can be used as indicators to investigate research fronts and predict research trends. The explosions in the literature were detected using CiteSpace software (Table 6). Due to environmental and sustainability concerns, this century has witnessed remarkable achievements in green technologies in materials science by developing biocomposite materials. The development of high-performance materials and natural resources is increasing worldwide. As we all know, there has been much interest in natural biopolymers recently and much acting. As demonstrated in Table 6, “dissolution,” “polylactic acid,” “regeneration,” “strength,” “antibacterial,” “efficient,” “cassava starch,” “bioactive compound,” “thermal stability,” “pretreatment,” “chitosan film,” and “nanocrystalline cellulose,” these 12 explosive keywords have emerged in recent years, and will continue to explode in 2021. It can be speculated that these keywords will become future research hotspots. Moreover, from these 12 keywords, it can be inferred that scholars and institutions engaged in the field of natural biopolymers in the future will shift from a single field to a more diversified research direction, focusing on “physical and chemical properties of natural biopolymers,” “natural biopolymers,” performance mechanisms of biopolymers,” “derivatives of natural biopolymers,” and “positive effects of natural biopolymers on human living standards."

**TABLE 6:** Top 25 keywords with strongest citation bursts.

Keyword	Year	Strength	Begin	End	2000–2021
Dissolution	2000	3.51	2017	2021	▂▂▂▂▂▂▂▂▂▂▂▂▂▂▂▂▂▃▃▃▃▃
Polylactic acid	2000	4.22	2018	2021	▂▂▂▂▂▂▂▂▂▂▂▂▂▂▂▂▂▂▃▃▃▃
Regeneration	2000	4.15	2018	2021	▂▂▂▂▂▂▂▂▂▂▂▂▂▂▂▂▂▂▃▃▃▃
Strength	2000	3.94	2018	2021	▂▂▂▂▂▂▂▂▂▂▂▂▂▂▂▂▂▂▃▃▃▃
Antibacterial	2000	5.81	2019	2021	▂▂▂▂▂▂▂▂▂▂▂▂▂▂▂▂▂▂▂▃▃▃
Efficient	2000	4.06	2019	2021	▂▂▂▂▂▂▂▂▂▂▂▂▂▂▂▂▂▂▂▃▃▃
Cassava starch	2000	4.02	2019	2021	▂▂▂▂▂▂▂▂▂▂▂▂▂▂▂▂▂▂▂▃▃▃
Bioactive compound	2000	3.62	2019	2021	▂▂▂▂▂▂▂▂▂▂▂▂▂▂▂▂▂▂▂▃▃▃
Thermal stability	2000	3.62	2019	2021	▂▂▂▂▂▂▂▂▂▂▂▂▂▂▂▂▂▂▂▃▃▃
Pretreatment	2000	3.30	2019	2021	▂▂▂▂▂▂▂▂▂▂▂▂▂▂▂▂▂▂▂▃▃▃
Chitosan film	2000	3.21	2019	2021	▂▂▂▂▂▂▂▂▂▂▂▂▂▂▂▂▂▂▂▃▃▃
Nanocrystalline cellulose	2000	3.21	2019	2021	▂▂▂▂▂▂▂▂▂▂▂▂▂▂▂▂▂▂▂▃▃▃

### 3.5 Emerging Research and Potential Value (2018–2021)

Since the scientific research results published in recent years have certain representativeness and reference value for the future research trend of a specific research field, we analyzed the research results related to natural biology in the Web of Science Core Collection (ScienceCitation Index Expanded, SCIE and Social Sciences Citation Index, SSCI) database from 2018 to 2021. Articles related to the polymer field are analyzed in depth. From 2018 to 2021, it is an explosive growth stage, with a total of 1,746 articles published, with an average annual publication volume of about 437 articles, 76,852 citations, and an average annual citation of about 19,213 times. Visible, natural research in biopolymers has become the focus of current and future scholars and institutions: research hotspots and research trends. Therefore, in order to understand the emerging research and potential value at this stage, only the articles on natural biopolymers from 2018 to 2021 were screened and summarized into three main research directions: “medical field,” “biochemistry field,” and “food science field."

#### 3.5.1 Medical Field

Since 2018, the application of natural biopolymers in the medical field has mainly been used in drugs; for example, rubber latex from the natural rubber tree has good biocompatibility and has been shown to act as a controlled drug by enhancing the process of angiogenesis, guiding, and recruiting cells responsible for osteogenesis. The released solid matrix induces tissue repair (Guerra et al., 2021). In addition to being used for drugs, natural biopolymers can also be used as carriers for medical machinery (John and Shaiba, 2019). Zhang W. et al. (2021) have shown that electrospun poly (caprolactone) scaffolds based on natural biopolymers can be used in bone, cartilage, and skin, The design and practice of tendon, ligament, and nerve fields (Durand et al., 2010) have efficient applicability, with potential value for future medical research and clinical applications; Bouhlouli et al. (2021) showed that the latest trends in bacterial cellulose (BC) applications are polymers. As scaffolds for different types of tissues, including bone, blood vessels, skin, and cartilage; Arango et al. (2021) studies have shown that sericin from natural silk is beneficial to the properties of biological keratinocytes and fibroblasts, making it become a biomaterial for repairing epithelial tissue, mainly used as a wound dressing and skin regeneration. Due to the environmental protection, low price, and high stability of natural biopolymers, scientists have performed the most research on them in the medical field. The field will continue to innovate and develop rapidly in the future.

#### 3.5.2 Biochemistry Field

Since 2018, the application of natural biopolymers in biochemistry has been mainly as proton conductors in bioelectronics (Jia M. P. et al., 2021). For example, as a natural biopolymer, the nucleic acid has excellent programmability, biocompatibility, and specific characteristics and can be directly recognized in biosensors through molecular recognition, improving research efficiency (Zhang et al., 2020); through direct chemical modification, targeting proteins. The terminal carboxyl or amine residues, and exploring the effect of surface hydrophobicity on proton conduction, can hinder the proton conduction of natural biopolymer proteins (Mondal et al., 2020). Xiong et al. (2020), based on light phenomena found in nature in biophotonic structures, integrated synthetic optically active nanocomponents into an organized hierarchical biopolymer framework for increasing optical functionality. There are few studies on the biochemical field of natural biopolymers. Most studies only use some single characteristics of natural biopolymers. However, the research on the combination of the characteristics of different natural biopolymers and their application in crop and plant breeding needs to be further strengthened in the future.

#### 3.5.3 Food Science Field

Since 2018, the application of natural biopolymers in food science has been mainly used in food, packaging, preservation, and coloring. For example, the flavonoid derivative stilbene is a flavonoid compound in several edible plants, which is often widely used as a food and food supplement (Grochowski et al., 2018). The bitterness of pea and potato protein was adjusted by compound coagulation (Zeeb et al., 2018). Natural biopolymer aromatic plant essential oil (EO) can be used as an excellent antibacterial agent, and its derivative EO microcapsule also has a particular fresh-keeping effect on fruits and vegetables (Guo et al., 2021). Edible packaging produced by processing fruit waste can prolong the shelf life of food and maintain its nutrition (Yadav et al., 2021). With the improvement of people’s living standards, the requirements for food colorants tend to be more natural colorants, such as carotenoids, betaine, anthocyanins, and chlorophyll (Alizadeh-Sani et al., 2020). As a famous natural colorant, curcumin has attracted extensive attention because of its excellent functional properties and the ability to change color with the change of pH value (Roy et al., 2022). The development of natural biopolymers is getting closer and closer to people’s daily life. With the development of global concepts such as sustainability, low carbon, green and environmental protection, degradable natural biopolymers have gradually replaced traditional rugged degradable plastic packaging bags, and natural biopolymers used in food will be a hot topic of research in the future.

## 4 Conclusion

The number and complexity of studies on natural biopolymers make it challenging to get a clear and comprehensive view of the rapid growth of information in the scientific literature. The development of natural biopolymers seems to be a beautiful way to mine the regularity of their changes. Bibliometrics is a transformative approach emerging as an emerging topic in the discipline. This study develops a data-driven strategy and framework for bibliometric analysis and presents research progress related to natural biopolymers from 2000 to 2021. The findings suggest that the field of natural biopolymers is attracting more interest from researchers worldwide, as evidenced by the massive growth in publications since 2000. The United States has published the most articles in this field, with 602, indicating that the research in this field is representative and authoritative, and China ranks second with a total of 538 articles, showing great potential and growth.

Comparing the performance of countries using the activity index (AI) and attractive index (AAI) shows that since 2000, China, Iran, and India have made significant progress in natural biopolymer research, becoming important new contributors. Since 2019, their influence has exceeded the global average, which means that the role of developing countries is more important than in the past, and their scientific research, technological and financial capabilities have begun to improve.

The top three discipline categories of natural biopolymer research are Polymer Science, Chemistry Multidisciplinary, Biochemistry, and Molecular Biology. The institution with the most significant number of published articles is the League of European Research Universities Leru, with a total of 119; five of the top 10 journals are from the United States, which shows that the United States has a high level of research in this field. “Physical and chemical properties of natural biopolymers,” “natural biopolymers,” performance mechanisms of biopolymers,” “derivatives of natural biopolymers,” and “positive effects of natural biopolymers on human living standards” are all emerging hotspots in the field of natural biopolymers.

2018 is the starting point for the explosive growth of the number of articles. Since 2018, the research of natural biopolymers has become the focus of current and future scholars and institutions. The researchers are mainly in the “medical field,” “biochemistry field,” and “food science field.”

This study will help researchers quickly identify their general patterns. Readers can gain exciting information from the rich bibliometric data. This study still has some limitations; for example, the data for natural biopolymers are only from the WOSCC database, which may not include other citation information, and although we identified the main research hotspots and their evolution, more in-depth information on each research hotspot, such as methodology, theoretical background and main findings of each work is still needed. Despite the aforementioned limitations, the findings of this study are based on objective data, are stable and reliable, and are generally unaffected by empiricism. This study can offer some suggestions for scholars interested in identity recognition to help them understand developmental trajectories and statistical models. Furthermore, an exciting avenue for those working in the future is to conduct more detailed analyses of natural biopolymers and discuss the results in greater depth: collaborations across multiple countries, institutions, and fields; comparisons between different natural biopolymers; the physical and chemical properties of natural biopolymers are improved and used; the operation and instruments for the modification of natural biopolymers and the development of derivatives of natural biopolymers in agriculture, ecology, soil, and other fields.
