# Supplementary material for: Global Trends in Natural Biopolymers in the 21st Century: A Scientometric Review
Source: Front Chem. 2022 Jul 7;10:915648. doi: 10.3389/fchem.2022.915648 (PMC9302608; doi:10.3389/fchem.2022.915648)
Supplement: Supplementary file 1 [file DataSheet1.docx]

Supplementary Material

# Supplementary Tables

| **AI** | **USA** | **China** | **India** | **Germany** | **Iran** | **Italy** | **France** | **Spain** | **Brazil** | **UK** |
| --- | --- | --- | --- | --- | --- | --- | --- | --- | --- | --- |
| 2000 | 1.16 | 0.86 | 0.50 | 0.83 | 0.00 | 0.00 | 0.99 | 0.00 | 2.80 | 0.00 |
| 2001 | 1.66 | 0.35 | 0.41 | 0.68 | 0.00 | 0.92 | 1.61 | 0.00 | 1.15 | 0.00 |
| 2002 | 1.74 | 0.29 | 0.33 | 1.65 | 0.00 | 0.00 | 1.32 | 0.00 | 0.93 | 0.00 |
| 2003 | 1.04 | 1.17 | 0.45 | 2.23 | 0.00 | 0.00 | 2.66 | 0.00 | 0.00 | 1.13 |
| 2004 | 1.14 | 0.68 | 0.00 | 0.32 | 0.00 | 1.32 | 1.93 | 0.00 | 0.55 | 0.98 |
| 2005 | 1.41 | 0.16 | 0.56 | 1.86 | 0.44 | 0.85 | 1.48 | 1.72 | 0.53 | 0.94 |
| 2006 | 1.31 | 0.45 | 0.00 | 0.57 | 0.40 | 0.78 | 3.07 | 1.19 | 1.46 | 0.87 |
| 2007 | 1.60 | 0.43 | 0.50 | 0.83 | 0.88 | 1.13 | 0.99 | 0.57 | 0.00 | 1.26 |
| 2008 | 1.27 | 0.74 | 0.85 | 1.21 | 0.28 | 1.10 | 1.44 | 1.40 | 0.34 | 0.61 |
| 2009 | 0.73 | 0.45 | 1.56 | 0.87 | 1.71 | 2.36 | 2.06 | 0.72 | 0.29 | 1.31 |
| 2010 | 1.43 | 0.61 | 0.96 | 1.75 | 0.62 | 1.59 | 1.04 | 0.20 | 0.99 | 0.44 |
| 2011 | 1.07 | 0.63 | 1.12 | 0.53 | 0.00 | 1.63 | 1.27 | 1.29 | 1.58 | 0.20 |
| 2012 | 1.23 | 0.63 | 0.80 | 0.97 | 0.85 | 0.66 | 1.15 | 1.51 | 0.82 | 1.29 |
| 2013 | 1.08 | 0.62 | 1.13 | 1.59 | 1.12 | 0.68 | 0.83 | 1.93 | 1.01 | 1.36 |
| 2014 | 0.62 | 0.88 | 1.21 | 1.09 | 0.59 | 1.03 | 1.10 | 0.93 | 1.14 | 1.53 |
| 2015 | 1.31 | 0.87 | 1.00 | 0.95 | 0.78 | 1.30 | 1.42 | 1.32 | 1.21 | 1.08 |
| 2016 | 0.83 | 1.43 | 0.85 | 0.67 | 0.84 | 0.51 | 0.44 | 0.93 | 1.13 | 0.68 |
| 2017 | 0.80 | 1.02 | 1.47 | 0.74 | 1.99 | 0.42 | 1.02 | 0.85 | 1.04 | 1.02 |
| 2018 | 0.91 | 1.27 | 0.90 | 0.87 | 1.39 | 1.19 | 0.41 | 1.13 | 0.88 | 1.32 |
| 2019 | 0.74 | 1.77 | 1.18 | 1.10 | 1.39 | 1.07 | 0.42 | 0.82 | 1.20 | 0.95 |

**Table S1.** The AI index for 10 countries

**Table S2.** The AII index for 10 countries

| **AAI** | **USA** | **China** | **India** | **Germany** | **Iran** | **Italy** | **France** | **Spain** | **Brazil** | **UK** |
| --- | --- | --- | --- | --- | --- | --- | --- | --- | --- | --- |
| 2002 | 1.33 | 0.18 | 0.08 | 0.13 | 0.00 | 0.49 | 0.85 | 0.00 | 4.38 | 0.00 |
| 2003 | 1.38 | 0.24 | 0.18 | 0.48 | 0.00 | 0.26 | 0.98 | 0.00 | 1.51 | 0.00 |
| 2004 | 1.54 | 0.27 | 0.35 | 0.74 | 0.00 | 1.02 | 0.70 | 0.00 | 1.98 | 0.55 |
| 2005 | 1.38 | 0.64 | 0.41 | 0.45 | 0.00 | 0.99 | 1.19 | 0.18 | 1.21 | 0.83 |
| 2006 | 1.39 | 0.54 | 0.17 | 0.62 | 0.00 | 0.87 | 1.63 | 0.47 | 0.94 | 0.68 |
| 2007 | 1.41 | 0.55 | 0.22 | 0.64 | 0.15 | 1.29 | 1.55 | 0.52 | 0.86 | 0.85 |
| 2008 | 1.22 | 0.58 | 0.39 | 0.66 | 0.16 | 1.00 | 1.82 | 0.86 | 0.69 | 0.97 |
| 2009 | 1.29 | 0.53 | 0.60 | 0.65 | 0.26 | 1.16 | 1.83 | 0.81 | 0.65 | 0.87 |
| 2010 | 1.29 | 0.46 | 0.72 | 0.73 | 0.33 | 1.11 | 1.88 | 0.77 | 0.54 | 0.99 |
| 2011 | 1.34 | 0.43 | 0.84 | 0.75 | 0.44 | 1.21 | 1.69 | 0.69 | 0.69 | 0.79 |
| 2012 | 1.23 | 0.41 | 0.81 | 0.79 | 0.48 | 0.97 | 1.64 | 0.78 | 0.67 | 0.84 |
| 2013 | 1.18 | 0.47 | 0.85 | 1.02 | 0.56 | 0.92 | 1.40 | 0.82 | 0.93 | 0.87 |
| 2014 | 1.10 | 0.53 | 0.86 | 1.14 | 0.54 | 0.85 | 1.32 | 0.99 | 0.88 | 1.00 |
| 2015 | 1.06 | 0.68 | 0.96 | 1.12 | 0.47 | 0.94 | 1.30 | 1.05 | 0.92 | 1.07 |
| 2016 | 1.12 | 0.76 | 1.01 | 1.13 | 0.59 | 0.84 | 1.15 | 0.99 | 0.88 | 0.95 |
| 2017 | 1.03 | 0.82 | 0.97 | 1.20 | 0.77 | 0.79 | 1.10 | 1.09 | 1.08 | 1.05 |
| 2018 | 0.98 | 0.88 | 1.09 | 1.15 | 0.88 | 0.83 | 1.01 | 1.11 | 1.09 | 1.07 |
| 2019 | 0.92 | 1.07 | 1.12 | 1.03 | 1.31 | 0.91 | 0.86 | 1.01 | 1.01 | 1.09 |
| 2020 | 0.88 | 1.31 | 1.08 | 0.99 | 1.47 | 1.02 | 0.68 | 1.10 | 1.05 | 1.12 |
| 2021 | 0.83 | 1.58 | 1.13 | 0.92 | 1.53 | 1.27 | 0.57 | 1.06 | 1.15 | 0.93 |
